# Etiology, Epidemiology, Pathophysiology, Signs and Symptoms, Evaluation, and Treatment of Vitamin A (Retinol) Deficiency

**DOI:** 10.7759/cureus.49011

**Published:** 2023-11-18

**Authors:** Shraddha Patil, Udit M Zamwar, Abhay Mudey

**Affiliations:** 1 Endocrinology, Jawaharlal Nehru Medical College, Datta Meghe Institute of Higher Education and Research, Wardha, IND; 2 Community Medicine, Jawaharlal Nehru Medical College, Datta Meghe Institute of Higher Education and Research, Wardha, IND

**Keywords:** retinol malabsorption, night blindness, xerosis, vitamin a, retinol deficiency

## Abstract

Vitamin A, also known as retinol, is a non-water-soluble vitamin. Vitamin A is very important for the proper functioning of the human body. Retinol, especially in the form of retinyl ester, can be found in many animal-based products and is essential for the efficient operation of many physiological processes. Fruits and vegetables are also excellent sources of vitamin A; the majority of them include carotenoids, which are precursors to vitamin A. The human body has the ability to convert natural retinols like retinyl ester, retinoic acid, and provitamin A into biologically active forms that interact with a variety of molecular targets like nuclear receptors and retinal opsins. This review article provides knowledge regarding retinol deficiency in humans. It provides brief information about the sources, etiology, epidemiology, pathophysiology, and treatment of vitamin A deficiency.

## Introduction and background

Vitamin A (Retinol) plays an important role in cell growth, metabolism, maintaining immunity, sight, and reproduction [1,2]. Retinol deficiency is a very common health issue found all around the globe, with high rates of mortality and disability, especially among young children in poor countries around the world. Poor absorption of retinol results in its deficiency and compromise of important physiologic processes. Retinol is naturally found in dark leafy green vegetables, milk, liver, fish, and other dairy products [3]. Retinol gets absorbed into the duodenum through hydrolysis by the pancreatic enzymes and emulsification with fats along with bile [4]. Most of the vitamin A is reserved in the liver cells, but a significant amount is also reserved in the adipose tissues and pancreas [5,6].

The Institute of Medicine (IOM) recommends that people should consume the recommended dietary allowance (RDA) of retinol to prevent deficiency. Healthy women should consume 700 mcg/day and healthy men should consume 900 mcg/day [7,8]. Children, pregnant females, and breastfeeding females should consume 300-400, 770, and 1300 mcg/day, respectively. For children aged 1-5 years, the minimum requirement is about 200 mcg/day to prevent symptoms of retinol deficiency. Levels of retinol in the serum are a good indicator of retinol deficiency. A retinol level of less than 20 mcg/dL indicates retinol deficiency [8]. The RDA for various age groups is depicted in Table 1 [7,8].

**Table 1:** Recommended dietary allowance for various people of different age groups

Category	Age Group	Recommended Dietary Allowance (mcg/day)
Infant	0-12 months	400-500
Children	1-8 years	300-400
Adult Males, Adult Females	9-70 years	800-900, 600-700 respectively
Pregnant Women	20-50 years	750-770
Lactating Mothers	20-50 years	1200-1300

Studies have shown that vitamin A deficiency-related ocular symptoms develop at retinol levels below 10 mcg/dL [8]. Retinol is consumed in the form of carotenoid or retinoid. Carotenoids are inactive variants of retinol, typically beta-carotenoids. Retinoids, on the other hand, are active variants of retinol. Examples include retinol, retinyl esters, and other retinoids. Studies have shown that retinoids have 75-100% absorption. Carotenoids, however, have a much lower absorption rate, which is largely determined by the carotenoid type and matrix of the food [9,10].

## Review

Methodology

**Figure 1:**
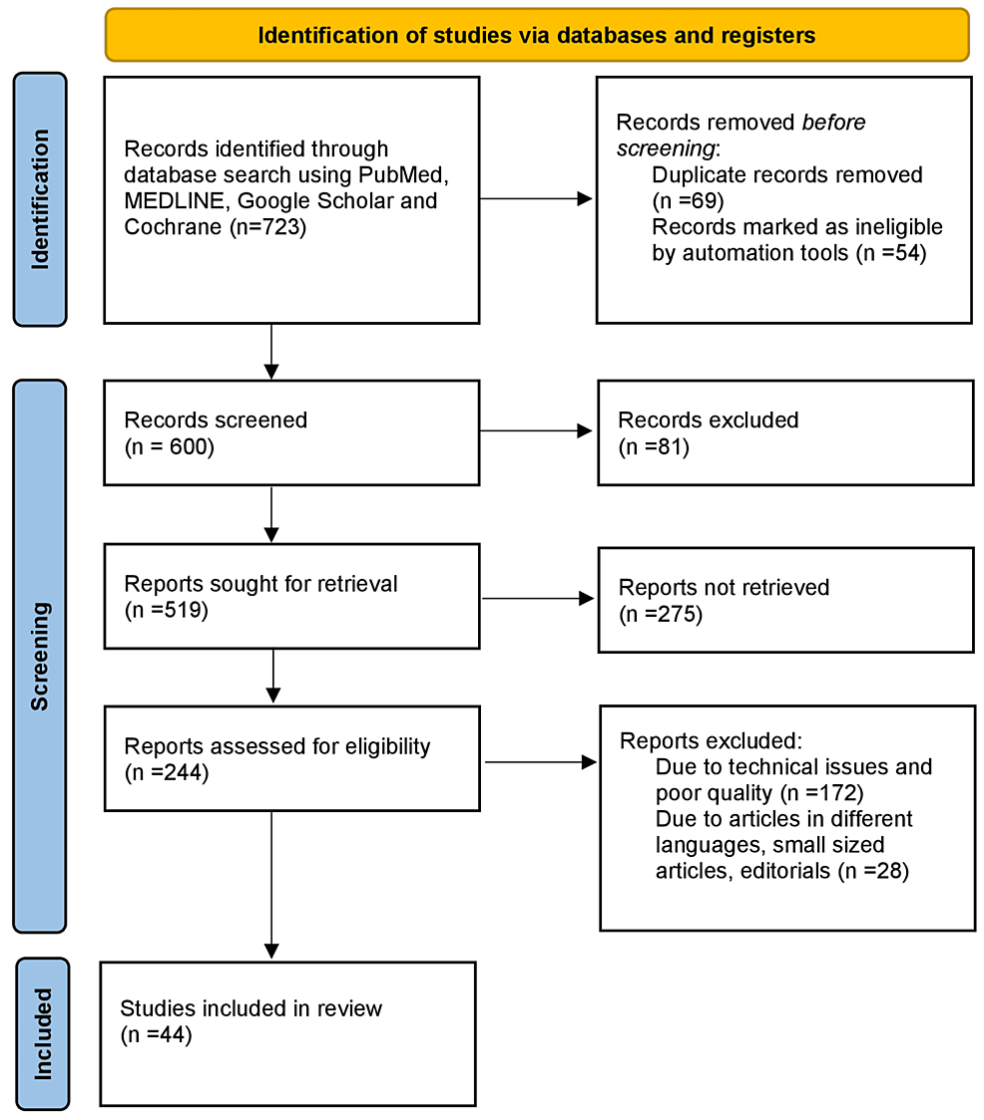
PRISMA methodology used in the study MEDLINE: Medical Literature Analysis and Retrieval System Online; PRISMA: Preferred Reporting Items for Systematic Reviews and Meta-Analyses

Using the electronic databases Medical Literature Analysis and Retrieval System Online (MEDLINE), Google Scholar, PubMed, and the Cochrane Library, a search of the articles published or translated into English was done. The query terms were "retinol" OR "vitamin A"; "epidemiology" OR "epidemiological data"; "prevalence" OR "incidence”; “risk factor” OR “etiology”; and "treatment” OR "modalities.” The articles included in this review focus on the studies conducted on retinol deficiency, the etiology, epidemiology, and pathophysiology of retinol deficiency, and treatment interventions. Studies conducted over the last 15 years have been included. The Preferred Reporting Items for Systematic Reviews and Meta-Analyses (PRISMA) method used in research methodology is depicted in Figure 1.

Etiology

In regions of the world with limited resources, inadequate nutrition, compounded by chronic inflammation from recurrent gastrointestinal infections, is the most common cause of retinol deficiency [11]. Adequate intake of retinol in children in these areas is frequently worsened by the co-occurrence of zinc (Zn) deficiency; Zn is necessary to absorb retinol and produce retinol-binding protein (RBP) [12]. Measles is an endemic disease in such areas and has been demonstrated to cause a rapid decrease in vitamin A levels of more than 30 percent [13]. This decrease in retinol levels leads to a decrease in RBP synthesis, resulting in high levels of retinol excretion in urine.

The mother's nutritional status has a significant effect on the quantity of vitamin A found in breast milk. The average vitamin A concentration in breast milk is enough to meet the infant's minimum daily requirement in places with scarce resources. This, however, prevents the buildup of hepatic reserves, which might result in elevated retinol levels soon after weaning [14]. Retinol deficiency, on the other hand, is incredibly uncommon in industrialized nations because of the accessibility of foods high in vitamin A, increased cleanliness, improved water supply, and improved health care. In affluent nations, numerous primary and secondary gastrointestinal malabsorption diseases are typically to blame for retinol-deficient occurrences.

The primary causes of retinol deficiency in developed countries are the various diseases or conditions affecting the intestine, liver, and pancreas. Inflammatory bowel disease (IBD), similar to the common gastrointestinal infections observed in developing countries, leads to an inflamed gastrointestinal mucosal layer, which, when combined with reduced retinol consumption orally, can result in deficient retinol content [15]. Liver diseases of any kind have been linked to retinol deficiency, although the exact mechanism of the deficiency is not fully understood. Possible explanations include a decrease in bile acid production required for absorption, and alterations in storage patterns [6].

Pancreatic insufficiency can lead to retinol deficiency due to inadequate exocrine function and the inability to produce the hydrolases necessary for absorption [16]. As a result, bariatric surgeries that bypass the duodenum to prevent fat absorption can result in a lack of absorption of retinol. Premature newborns are particularly vulnerable to retinol deficiency due to their immature gastrointestinal tract, which does not provide enough amount of absorption of retinol, limited stores of retinol, and increased requirements during a period of rapid growth [17].

Epidemiology

Globally, the majority of vitamin A deficiencies are observed in children under the age of five in developing nations. Although global estimates of vitamin A deficiency among young children are declining, they have been estimated to be as high as 30% among children under five and account for approximately 2% of mortality in them [18]. Furthermore, pregnant and breastfeeding women are particularly vulnerable to vitamin A deficiency due to their increased daily needs. A study conducted in rural Ethiopia in 2019 revealed that 76% of pregnant and lactating mothers had vitamin A deficiency [19]. Retinol deficiency has not been associated with a gender preference [20,21].

The prevalence of retinol deficiency in the general population of the United States (US) in 2013 was estimated to be 0.3%. However, it is important to note that the prevalence of vitamin A toxicity is much higher than that of deficiency. Symptoms of vitamin A deficiency typically involve a malabsorption process or a restrictive diet. In the US, 16% of children with IBD are estimated to be vitamin A deficient at diagnosis. Crohn’s disease is more prevalent in children in the US than ulcerative colitis. Around 70% of patients with hepatitis C eligible for transplantation are vitamin A deficient, and there is a positive relationship between cirrhosis severity and prevalence of retinol deficiency [22,23].

Brief pathophysiology, signs and symptoms, and evaluation

Pathophysiology

Vitamin A plays a critical role in the production of visual pigment, the maintenance of mucosal membrane integrity, and the functioning of the immune system. A deficiency of vitamin A can result in night blindness, which is caused by a deficiency of retinal pigment in the retina. If the deficiency persists, the retinal rods will deteriorate, resulting in xerophthalmia, which in turn will lead to true blindness [24-26]. Xerosis and breakdown of the intestinal and pulmonary mucosal membranes, combined with immune dysfunction, result in recurrent infections and anemia due to chronic inflammation [27].

Signs and Symptoms

Patients with a well-documented medical history may be suspected of vitamin A deficiency. Malabsorptive processes, such as IBD, chronic gastrointestinal infection, cirrhosis, insufficiency of the pancreas, measles, and pregnant or breastfeeding women should be a cause for concern [28,29]. The most common manifestation of chronic retinol deficiency is the gradual onset of night blindness, an increased incidence of gastrointestinal, lung, and urine infections, as well as the development of xeroderma and phrynoderma [30,31].

As the deficiency progresses, Bitot spots, a type of triangular white foamy lesions on the conjunctiva, may appear. If retinol deficiency persists, it may manifest itself in the form of corneal xerosis, ulceration, and, eventually, keratomalacia, as the ulceration heals, resulting in scarring and eventual blindness. When any infection happens (especially measles), patients with an acute deficiency may develop corneal exudation without prior night blindness, Bitot spots, or phrynoderma [26]. However, this condition is not exclusive to retinol deficiency and has been linked to other nutritional deficiencies [31].

Evaluation

Classical exam findings, as well as confirmatory laboratory testing, can be used for the diagnosis of vitamin A deficiency clinically. Xerophthalmia, for example, is almost pathognomonic of vitamin A deficiency [32]. For patients with a less apparent history and physical examination, the provider may order a serum retinol test. Serum retinol levels are typically defined as below 20 mcg/dL, but serum retinol concentrations may be normal even if total body storage is low, as retinol levels in the liver are maintained by circulating stores [8]. Quantification of liver retinol levels on biopsy is the gold standard for assessing total body vitamin A [33]. However, due to the associated risks, liver biopsy is not performed frequently to evaluate the retinol levels.

Treatment

Vitamin A supplementation (VAS) is a treatment for retinol deficiency. Numerous studies have demonstrated that vitamin A supplementation in retinol-deficient patient populations has been associated with a reduction in childhood mortality and morbidity [34]. Retinol deficiency has been shown to make a clinically meaningful difference in patients with a serum retinol concentration below 20 mcg/dL. Patients with retinol levels above 30 mcg/L do not benefit from vitamin A supplementation and should adhere to the RDA [35].

In areas with a high retinol deficiency prevalence, the WHO has proposed a single-dose universal VAS for the selected populations. This includes a one-dose (1 lakh IU) for children of the age group of six to eleven months, followed by two-dose (2 lakh IU) regimens every four to six months until the age of five years [36]. As fetotoxicity is a matter of concern, pregnant women at risk of retinol deficiency should be supplemented at lower doses; the recommended dosage is 10,000 IU per day or 25,000 IU per week for 12 weeks [37]. The WHO has discontinued its universal supplementation recommendations for children under the age of six months or post-partum women [38-40].

The international guidelines do not prescribe a specific dosage for VAS in cases of asymptomatic vitamin A deficiency in regions with access to adequate resources. Instead, the dosage of vitamin A supplementation is determined by the severity of the deficiency. The WHO recommends that children under six months of age should receive 50,000 IU of VAS for xerophthalmia, children of the age group of six to twelve months should receive 1 lakh IU, and children above the age of 12 months should receive 2 lakh IU daily per day for two days. An additional dose should be administered after 2 weeks. The WHO recommends the same dosage for any patient with severe cases of measles, regardless of whether they are suffering from retinol deficiency or not [41]. As for particular vitamin A supplementation strategies, patients with Zn deficiency have an inadequate response to VAS and should be treated concomitantly with Zn supplementation [12].

In countries with access to resources, post-bariatric patients and newborns are subject to specific dosing guidelines. Patients after undergoing bariatric operation are advised to consume 10 thousand IU of vitamin A supplementation per day and to adjust dosage as necessary based on daily monitoring of serum vitamin A. In some cases, bariatric patients may require as much as 100,000 IU VAS per day [42]. As for preterm infants, there are currently no guidelines in place. However, research has demonstrated that VAS administered at a rate of 10,000 IU per day for four weeks in very premature newborns has been associated with significant results, including a 56% reduction in all-cause mortality, a reduction in oxygen requirement, a decrease in sepsis and post-operative drug administration (PDA), and a decrease in hospital stay [43]. When a dose of 1500 IU was supplemented daily in extremely premature newborns, it resulted in a significant reduction in the incidence of retinopathy. Additionally, a decrease of almost 50% in bronchopulmonary lung dysplasia was observed [44]. Retinol deficiency related to other malabsorption processes is managed on an individual basis.

Retinol should be consumed strictly according to the RDA. Consumption of excess retinol for a long time can lead to chronic intoxication. Chronic intoxication is manifested by dry skin, cheilosis, glossitis, vomiting, alopecia, bone demineralization and pain, hypercalcemia, lymph node enlargement, hyperlipidemia, amenorrhea, and increased intracranial pressure. Chronic intoxication with vitamin A can lead to the development of liver fibrosis and portal hypertension. When taking vitamin A above the permissible norm, pregnant women may develop spontaneous abortion and damage to the fetus with disorders of the craniofacial and cardiac valve apparatus.

Findings from the different studies are included in Table 2.

**Table 2:** Findings of studies included in this review article

Author Name	Year	Findings
Harrison EH [4]	2012	Explores the various mechanisms and enzymes that aid in the absorption of vitamin A and its derivatives in the intestine. Molecular mechanisms involved in the digestion of vitamin A are reviewed. Molecular pathways involved in the absorption of carotenoids and retinol by the intestine are reviewed. It concludes that many proteins are involved in the regulation of the absorption of retinol and carotenoids by the intestine.
Miller M, et al. [8]	2002	Discusses the various causes of retinol deficiency in children and their mothers, and the recommended dietary allowance (RDA) of retinol to prevent deficiency. It concludes that children at a young age face deficiency of retinol mainly due to their retinol-deficient mothers and so these mothers produce breast milk that has a low amount of retinol, and their diets contain very little amount of retinol. It also concludes that vitamin A supplementation is very important to treat retinol deficiency effectively.
Reboul E [9]	2013	Explores and focuses on the various transport proteins that aid the transport of vitamin A and its derivatives. It concludes that some particular proteins like cytosolic cellular retinol-binding protein II (CRBPII), and several non-specific transporters have been identified, and other transporters like the cobalamin apical membrane transporter remain to be identified.
Rahman MM, et al. [12]	2002	Investigates the correlation between zinc and vitamin A deficiency, and the necessity of zinc supplementation in vitamin A-deficient children to increase vitamin A absorption. The study concludes that supplementing zinc and cobalamin in combination improves the levels of retinol in retinol-deficient children.
West CE [13]	2000	Discusses the importance of vitamin A supplementation in people suffering from measles. This study concludes that retinol supplementation improves immunity and helps in better recovery from measles.
Kositamongkol S, et al. [17]	2011	Explores the nutritional status of retinol in very-low-birth-weight infants and the need for supplements in them. The study concludes that high incidences of retinol and vitamin E deficiency have been found in very-low-birth-weight infants starting from birth to term postmenstrual age. So these infants should be treated with a higher dose of retinol supplementation.
Saari JC [25]	2016	Explores the importance of vitamin A in the maintenance of optimum vision. This study concludes that a deficiency of retinol causes many ocular manifestations and should be treated early when diagnosed.
Gilbert C [26]	2013	Discusses the various ocular signs and manifestations of retinol deficiency. This study concludes that retinol deficiency can cause various ocular manifestations like night blindness, xerosis of conjunctiva and cornea, Bitot's spots, etc.
Surman SL, et al. [30]	2020	Discusses the consequences of chronic vitamin A deficiency on immunity, cells, and various organ systems. This study concludes that chronic retinol deficiency has a huge impact on the patients' health and various organs.
Maronn M, et al. [31]	2005	Investigates whether phrynoderma is a manifestation of vitamin A deficiency or not. This study concludes that phrynoderma is a type of follicular hyperkeratosis and is related to retinol deficiency, and may present as a clinical manifestation.
Tanumihardjo SA [33]	2011	Explores the importance of various biomarkers for the detection of retinol deficiency. This article concludes that consuming the required amount of retinol is a necessity. Various biomarkers play a major role in detecting retinol deficiency in the body.
Imdad A, et al. [34]	2017	Discusses the importance of the amount of vitamin A to be supplemented in the children to prevent or reduce morbidity and mortality. This article concludes that vitamin A supplementation is a necessity in children. It helps to boost their immunity and enhances resistance to diseases. This reduces the morbidity and mortality rate of children.
[36]	2011	Provides guidelines for the amount of vitamin A to be supplemented in infants and children from 6 to 59 months of age. These guidelines conclude that retinol supplementation is very important in infants and children from 6 to 59 months of age as a public health intervention to reduce their mortality and morbidity.
[37]	2011	Provides guidelines for the amount of vitamin A to be supplemented in the pregnant female population. These guidelines conclude that retinol supplementation is not required to be given compulsorily in the antenatal care period. It should only be supplemented in women who live in areas that are endemic to retinol deficiency.
[38]	2011	Provides guidelines for the amount of vitamin A to be supplemented in infants from 1 to 5 months of age. These guidelines conclude that retinol supplementation is not a necessity in infants from 1 to 5 months of age as a public health intervention to reduce their morbidity or mortality and breastfeeding is enough to meet the retinol requirements.
[39]	2011	Provides guidelines for the amount of vitamin A to be supplemented in the neonates. These guidelines conclude that retinol supplementation is not a necessity in neonates as a public health intervention to reduce their morbidity or mortality and breastfeeding is enough to meet the retinol requirements.
[40]	2011	Provides guidelines for the amount of vitamin A to be supplemented in the postpartum female population. These guidelines conclude that retinol supplementation is not a necessity in postpartum women as a public health intervention and eating ample retinol-rich foods is enough to maintain the retinol levels in the body.

## Conclusions

Vitamin A (retinol) deficiency is very common all over the globe. It can be naturally gained by the body from fruits and vegetables like green leafy vegetables (spinach, lettuce), milk, liver, fish, etc. It plays an essential role in cell growth, metabolism, maintaining immunity, healthy eyesight, and reproduction. The RDA of vitamin A is different for males and females and also different for children, pregnant women, and lactating mothers. Vitamin A deficiency mainly results in ocular manifestations like night blindness, corneal xerosis, keratoconjunctivitis, etc. Retinol deficiencies can be caused due to various pathological conditions of the intestine, liver, and pancreas. The appropriate treatment for vitamin A deficiency is VAS. The WHO has provided various VAS dosages for people of different age groups. To conclude, vitamin A is an extremely necessary nutrient for the body and people should regularly keep a check on their vitamin A levels and look for any possible symptoms. People should include ample amounts of green leafy vegetables and other vitamin A-rich sources in their daily diet to keep their vitamin A levels in check as prevention is always better than cure. 
